# Lattice thermal conductivity of borophene from first principle calculation

**DOI:** 10.1038/srep45986

**Published:** 2017-04-04

**Authors:** Huaping Xiao, Wei Cao, Tao Ouyang, Sumei Guo, Chaoyu He, Jianxin Zhong

**Affiliations:** 1 Hunan Key Laboratory for Micro-Nano Energy Materials, Xiangtan University, Xiangtan 411105, Hunan, China

## Abstract

The phonon transport property is a foundation of understanding a material and predicting the potential application in mirco/nano devices. In this paper, the thermal transport property of borophene is investigated by combining first-principle calculations and phonon Boltzmann transport equation. At room temperature, the lattice thermal conductivity of borophene is found to be about 14.34 W/mK (error is about 3%), which is much smaller than that of graphene (about 3500 W/mK). The contributions from different phonon modes are qualified, and some phonon modes with high frequency abnormally play critical role on the thermal transport of borophene. This is quite different from the traditional understanding that thermal transport is usually largely contributed by the low frequency acoustic phonon modes for most of suspended 2D materials. Detailed analysis further reveals that the scattering between the out-of-plane flexural acoustic mode (FA) and other modes likes FA + FA/TA/LA/OP ↔ TA/LA/OP is the predominant phonon process channel. Finally the vibrational characteristic of some typical phonon modes and mean free path distribution of different phonon modes are also presented in this work. Our results shed light on the fundamental phonon transport properties of borophene, and foreshow the potential application for thermal management community.

Followed by the discovery of graphene^1^,^2^,^3^, the two-dimensional (2D) nanomaterials have attracted explosion of interest due to their extraordinary physical properties. For example, Dirac dispersion relation and remarkable carrier mobility are predicted in silicene^4^,^5^ and germanene^6^,^7^ (the congeners of carbon). Unlike the zero-bandgap in graphene, the 2D transition metal dichalcogenides (TMDCs)^8^,^9^ and phosphorene^10^,^11^ are semiconductor and predicted to exhibit excellent thermoelectric performance^12^,^13^. Moreover, impressive gas storage and molecular sieves are also reported in graphyne, a graphene allotrope containing both sp and sp^2^ hybridization states^14^. These unique 2D geometric structure and its corresponding superb physical properties foreshow the great potential for integration into the next-generation optoelectronic and energy storage and conversion devices.

Boron (B) is a neighbor of carbon in the periodic table. Its 2D allotrope, borophene, is also a hot focus in the current nanomaterial research community. Based on the density functional theory, researchers proposed numerous configurations of single-atom layer borophene and tested the corresponding stability of thermodynamics^15^,^16^. Numerous intriguing physical properties are predicted as well, e.g., the *Pmmn*-borophene has a distorted Dirac cone^17^, after the 2D tetrels nanostructure (graphene, silicene, and germanene) the second elemental 2D material with massless Dirac fermions. Meanwhile, the electronic property of borophene can be easily tuned by the substrate interactions or surface modifications^18^,^19^,^20^. On the other hands, the boron-boron bonds in borophene are nearly as strong as the carbon-carbon bonds in graphene^21^. That is to say, the borophene possess outstanding mechanical property as well^22^. With these remarkable properties, borophene will hold promise for possible applications ranging from electronic to photovoltaic devices. However, owning to the complex of bonding between boron atoms, 2D boron sheet or boron cluster is very difficult to realize in experiment. Recently, breakthroughs are made in such experiment^23^,^24^,^25^. A highly stable quasi-planar boron cluster B_36_, a potential basis of extended two-dimensional boron sheet, is synthesized by Piazza *et al*.^23^. Under ultrahigh-vacuum conditions, Mannix *et al*.^24^ fabricated atomically thin, crystalline 2D boron sheets (i.e., borophene) on silver surfaces. The experimental breakthroughs lay the foundation of the potential application of borophene in nanodevices. It is well known that the application of a material is also closely related to its thermal transport properties. For instance, in order to ensure the stability and extend the lifetime of electronic or photovoltaic devices, a high thermal conductivity is indispensable for removing the accumulated heat^26^. However, in the thermoelectric and thermal insulation community researchers are pursuing low thermal conductivity^27^,^28^. Therefore, it is necessary to understand the unexplored phonon transport properties of the new discovered borophene. On the other hands, compared with the superb thermal conductivity of suspended graphene (about 3500 W/mK)^29^, it is natural to ask how much the thermal conductivity of borophene could approach. How important is the contribution of each phonon branch to the overall thermal conductivity of borophene? What is the physical mechanism of the corresponding phonon propagation?

Inspired by these integrant needs, in this paper, we calculate the intrinsic lattice thermal transport in borophene by solving the phonon Boltzmann transport equation (PBTE) with interatomic force constants extracted from first-principle calculations. The value of thermal conductivity of borophene is predicted to be about 14.34 W/mK at room temperature, which is much lower than that of graphene and foreshows the potential application in thermal management. Meanwhile, through examining the phonon mode property, we found that unlike the large contribution by low frequency acoustic phonons of the thermal conductivity of graphene, some high frequency phonon modes dominate the thermal transport of borophene. Finally, the phonon mean free path (PMFP), which is an important parameter for the study of size effect and nanoengineering, is also calculated.

## Results and Discussion

**Figure 1:**
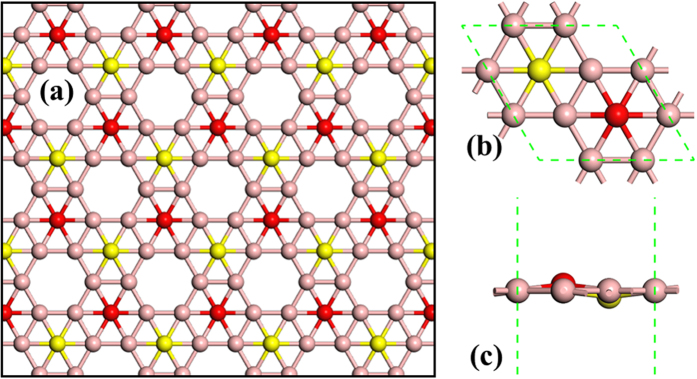
(**a**) Top view of atomic structure of borophene. (**b**) Top view and (**c**) side view of primitive cell of borophene. The yellow and red balls respectively denote the boron atoms move inward and outward from the plane.

In the present work, we mainly focus on the buckling alpha′(α′)-boron sheet, which is predicted to be more stable theoretically^15^. Here, we use borophene instead of α′-boron sheet for short and convenience. As shown in Fig. 1, the borophene is slightly buckled where every two adjacent boron atoms with coordination number 6 move inward (yellow balls) and outward (red balls) from the plane. The equivalent lattice constant of borophene is *a* = 4.37 Å, and the vertical distance from the plane is about ±0.173 Å. All these structure parameters obtained in this work agree reasonably with the previous theoretical work^15^.

**Figure 2:**
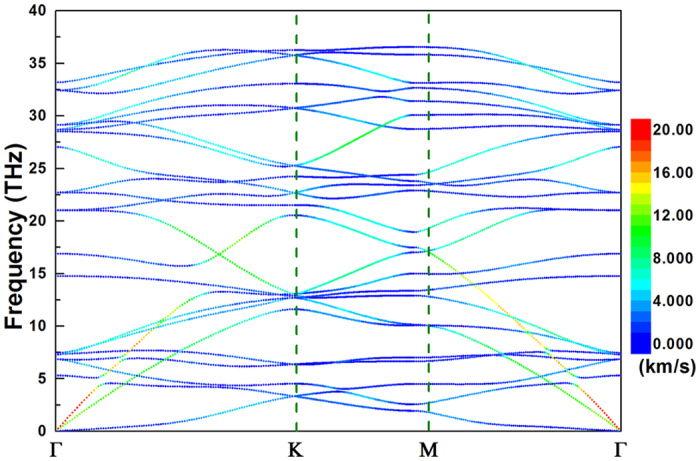
The phonon spectra of borophene along several high symmetry directions. The magnitude of group velocity of each phonon modes is projected to the corresponding phonon branches, which is denoted by the different color.

Based on this fully optimized geometric structure, the phonon dispersion spectrum of borophene is calculated through solving the eigenvalues of the 2^nd^ IFCs. One can see clearly from Fig. 2 that the phonon dispersion of borophene has no imaginary frequencies and the optical phonon branches have quite high eigenvalues (35.54 THz in borophene as compared with 47.98 THz in graphene). This suggests that the 2D boron sheet is dynamically stable and the bonding among boron atoms is almost as strong as carbon-carbon bonds in graphene. There exist 8 boron atoms in each primitive cell of borophene, implying the corresponding phonon spectrum possesses 3 acoustic and 21 optical phonon branches. The three lowest phonon branches around the Γ point are acoustic phonon branches, i.e. the out-of-plane flexural acoustic mode (FA), the in-plane transverse acoustic mode (TA), and longitudinal acoustic mode (LA). As we known that owning to the rapid decay of the transversal forces, the FA phonon mode of graphene has a quadratic dispersion with respect to the wave vector around the Γ point^30^,^31^. As for the borophene shown in Fig. 2, however, not only the TA and LA, but also the FA present linear dispersion versus the wave vector around the Γ point. This is mainly attributed to the intrinsic buckled geometric structure. Similar behavior has also been observed in silicene and phosphorene^30^,^32^,^33^, which is a general feature of 2D buckled materials. Moreover, in Fig. 2 the magnitude of group velocity of each phonon modes of borophene is also projected to the corresponding phonon branches, which is represented by different color. It can be found that the group velocity of TA and LA phonon branches is relatively high (~20 km/s), while that of FA is quite small (about 5 km/s). On the other hands, some optical phonon modes (frequency ranges from 10~20 THz) also possess large group velocity even comparable with that of the TA and LA branches. These unique harmonic phonon properties of borophene will play vital role on the thermal transport, which will be discussed in the following.

**Figure 3:**
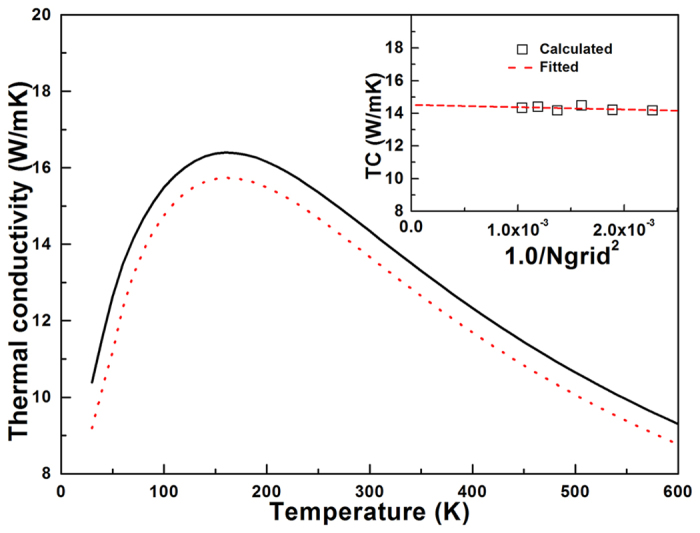
The total lattice thermal conductivity of borophene as a function of temperature by using the iterative (solid line) and relaxation time approximation (RTA) solution of PBTE. (Inset) The dependence of lattice thermal conductivity of borophene on the phonon *q*-grid using iterative approach (300 K).

With the confidence of the 2^nd^ and 3^rd^ IFCs, we calculate the lattice thermal conductivity of borophene through solving the PBTE (the van der Waals diameter of boron atom is taken as the thickness of borophene, ~4.16 Angstrom), and the results are depicted in Fig. 3. Owing to the intrinsic symmetry of the gemoetric structure, the thermal transport in borophene is isotropic (κ_*xx*_ = κ_*yy*_). Therefore, we only consider the thermal conductivity along the *x* direction (κ_*xx*_) in our calculation. The convergence of lattice thermal conductivity versus the density of phonon q-grid is tested firstly. As shown in the inset of Fig. 3, although the phonon q-grid increases from 17 × 17 × 1 to 31 × 31 × 1, the change of the thermal conductivity of borophene is quite slight (within less than 3% which representing the error in our calculation), presenting insensitive phonon q-grid effect on the thermal conductivity and also implying the convergence of the PBTE calculation. Therefore in order to keep the computation consistent we employ the 31 × 31 × 1 phonon q-grid for all of our calculations. One can find from Fig. 3 that the lattice thermal conductivity of borophene firstly increases with temperature ascending. After the temperature larger than 150 K, however, the conductivity decreases as the temperature increases. This is a common phenomenon presented in the lattice thermal conductivity of crystalline materials, which is originated from the intrinsic enhancement of phonon-phonon scattering with temperature increasing. At room temperature, the lattice thermal conductivity of borophene is about 14.34 W/mK, which is about two orders of magnitude smaller than that of suspended graphene (about 3500 W/mK)^29^ and foreshow the potential applications in thermal management community. Moreover, as a comparison, the thermal conductivity of borophene is also computed with relaxation time approximation (RTA) method. We note that both iterative and RTA method give similar values of thermal conductivity (e.g., the conductivity is about 13.67 W/mK by using RTA approach) and similar trends in the temperature dependence.

**Figure 4:**
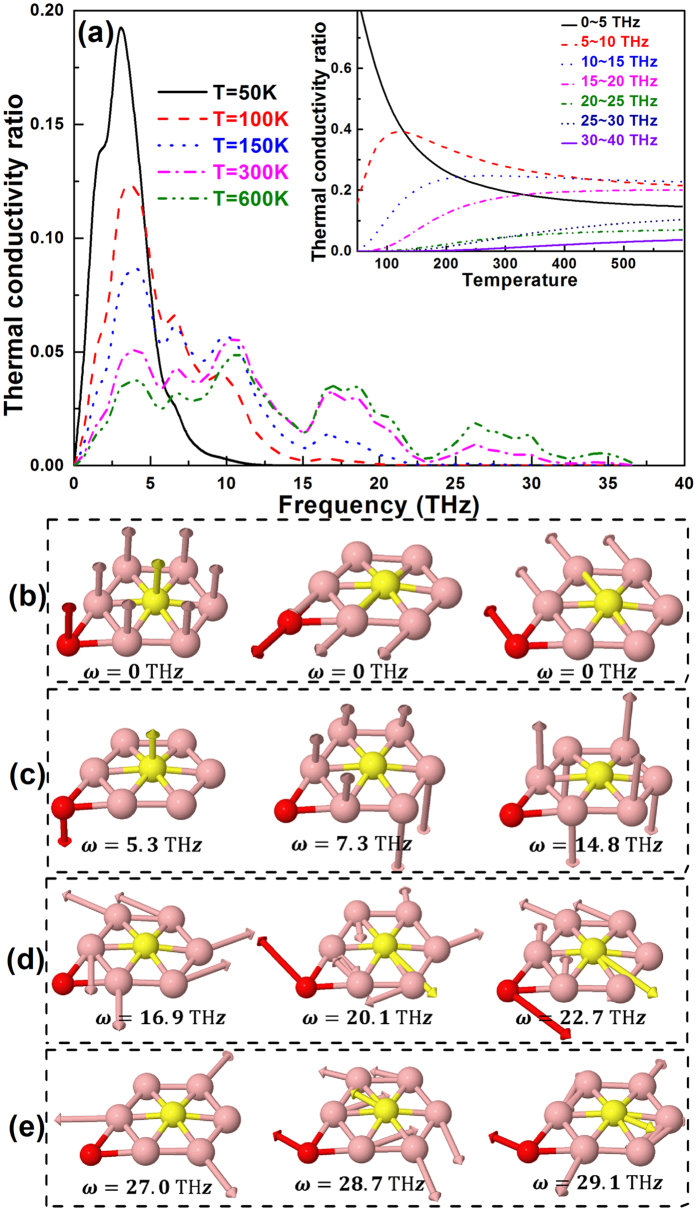
(**a**) The frequency dependent lattice thermal conductivity of borophene at five typical temperature. (Inset) The contribution of phonon modes with different frequencies to the total lattice thermal conductivity of borophene as a function of temperature. (**b**–**e**) Some typical phonon modes of borophene together with their frequencies at the Γ point. The arrows denote the atomic displacement directions.

Owing to the mixture of acoustic and optical phonon dispersion of borophene, it is quite difficult to distinguish the contribution from different phonon modes to the lattice thermal conductivity. Therefore, in this work we sort the phonon modes with different frequency, and analyze their contributions to the conductivity, as shown in Fig. 4(a). One could note that below 50 K, only the phonons with frequency small than 5 THz (mainly acoustic phonon modes) is propagated and contributed to the thermal conductivity. At higher temperature, nevertheless, the contributions from some high frequency phonon modes gradually become significant, especially for the phonons in the range of 5.0~15.0 THz, 16.0~23.0 THz, and 25.0~30.0 THz. From the inset of Fig. 4(a), we could see this behavior more clearly. As the temperature larger than 100 K, the phonon modes range from 5~20 THz gradually govern the total thermal conductivity of borophene. For instance, at room temperature the contributions from phonon modes with frequency range of 5~10 THz, 10~15 THz, and 15~20 THz, are about 27.8%, 27.6%, and 17.8% of the total thermal conductivity, respectively. Such phenomenon in borophene is quite different from the traditional 2D nanomaterials. Generally speaking, only low frequency acoustic phonon modes play critical role on the lattice thermal conductivity of 2D nanomaterials, such as suspended graphene^30^,^31^, silicene^30^, and phosphorene^32^, while the high frequency phonon modes always do not contribute much. In borophene, however, the interaction of optical phonon modes with TA and LA phonon modes and among themselves results in obvious band dispersion and higher group velocities (demonstrated in Fig. 2). This is one reason for the dominant contribution of thermal conductivity from some phonon modes with high frequency at higher temperature.

To describe the characteristic of these phonons directly, in Fig. 4(b–e), some typical lattice vibrations of borophene at the Γ point are presented. It is evidently that the three phonon modes shown in Fig. 4(b) from left to right respectively represent the vibration of FA, TA, and LA phonons. However, the phonon modes shown in Fig. 4(c–e) have optical character, i.e., each boron atoms move in opposite direction to each other. Meanwhile, one can also note that the phonons in different frequency region have different vibrational features. The boron atoms have out-of-plane motion as for the phonon modes in range 5.0~15.0 THz, while that have in plane motion for the phonons in range 16.0~23.0 THz and 25.0~30.0 THz. Moreover, an interesting intrinsic variation is observed at phonon modes with frequency 14.8 THz (the rightmost image of Fig. 4(c)). In this mode, the buckled boron atoms stay fixed, while each plane boron atom has an out-of-plane counter phase motion with respect to the neighboring boron atoms. Similar phonon modes could also be found at 5.3 THz (the leftmost image of Fig. 4(c)) and 16.9 THz (the leftmost image of Fig. 4(d)).

**Figure 5:**
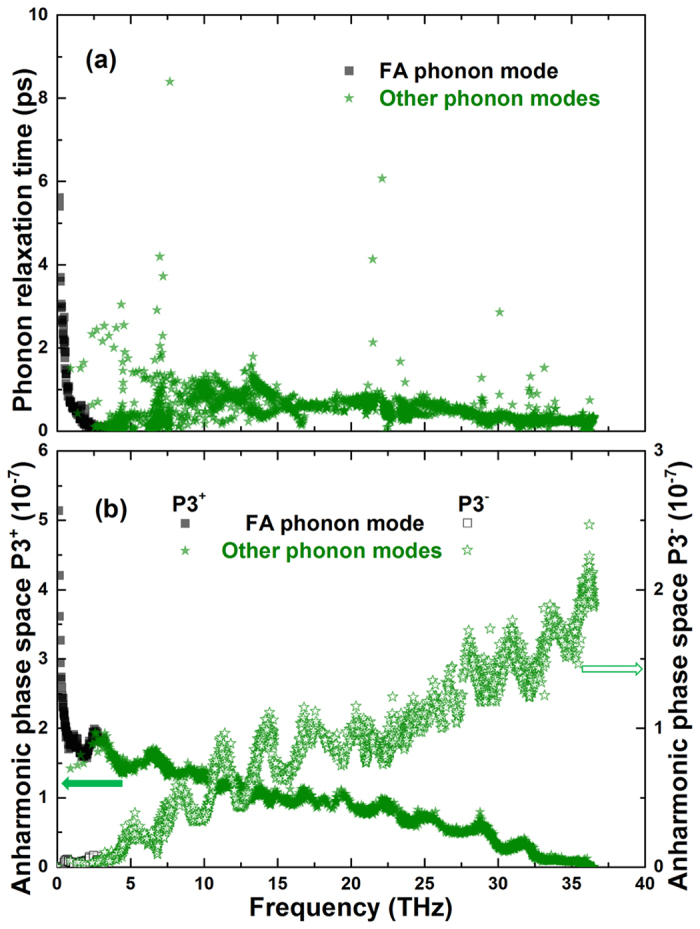
(**a**) The phonon relaxation time at room temperature (300 K) of different phonon modes as a function of frequency. (**b**) The frequency dependent anharmonic scattering phase space for absorption (P3^+^ solid symbols) and emission process (P3^−^ hollow symbols) of borophene at room temperature.

In order to find out more physical insight into the phonon mode properties, we extract frequency-dependent phonon relaxation time (*τ*) and anharmonic scattering phase space (P3) of borophene. It can be seen from Fig. 5(a) that the phonon relaxation time of FA is not significantly larger than other phonon modes, even smaller than the TA, LA, and some optical phonon modes. This is quite different from the case of graphene and mainly attributed to the broken of the symmetry-based phonon-phonon scattering select rule by the buckled structure of borophene. Therefore, the FA phonon mode has quite little contributions to the overall lattice thermal conductivity of borophene. Meanwhile, one can also find that some high frequency phonon modes have relatively large relaxation time compared to the low frequency acoustic phonon modes, especially for the phonon frequency around 7.5, 22.5, and 30 THz. This phenomenon can be explained by the evident frequency gap (energy gap, around the K point) among the phonon branches, which will strongly restrict the anharmonic phonon-phonon scattering due to the requirement of energy conservation. Considering the high frequency phonon modes also have large group velocity as demonstrated in Fig. 2, thus, it is easy to understand that these phonon modes contribute most to the overall lattice thermal conductivity. In Fig. 5(b), the anharmonic scattering phase space which could qualitatively characterize scattering channel of borophene is presented. Here, both absorption (P3^+^) and emission (P3^−^) processes are calculated. It is obvious that the P3^+^ of FA phonon mode is much larger than that of other phonon modes. Meanwhile, the P3^+^ (FA) is more intense than that the emission process P3^−^ (FA). That is to say, in the thermal transport of borophene the scattering processes like FA + FA/TA/LA/OP ↔ TA/LA/OP (the OP is abbreviation of optical phonon modes) dominate the scattering channels, and such processes occur more easily from left to right. This large phase space of FA modes is due to the broken of symmetry scattering select rule as discussed above. As for some high frequency phonon modes, the relatively large energy gap severely restricts the corresponding scattering phase space for three-phonon processes like 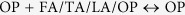, which gives rise to the large phonon relaxation time and predominant contribution to the lattice thermal conductivity of borophene.

**Figure 6:**
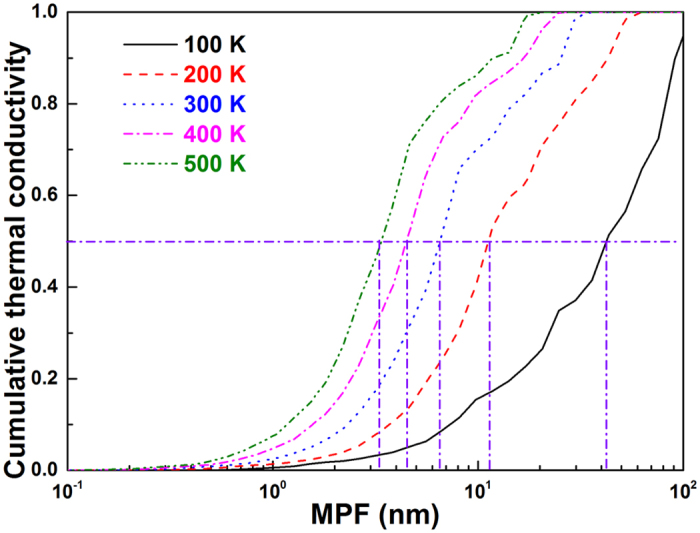
The accumulative lattice thermal conductivity of borophene as a function of phonon MFP at different temperature.

The accumulative thermal conductivity with respect to phonon mean free path (MFP) is helpful to predict the size effect of borophene. We also show this plot for borophene at different temperatures in Fig. 6. One can find that the MFPs corresponding to 50% accumulation conductivity of borophene at 100, 200, 300, 400, and 500 K are calculated to be about 41, 11, 6.4, 4.5, and 3.3 nm, respectively. This is quite different from the case of graphene, where the phonons with MFP longer than 1 um contribute nearly half of the thermal conductivity^29^,^30^. This suggests that the phonons of borophene will achieve diffusion transport when the size is larger than a few nanometers, implying the design of nanostructures (e.g., grain boundaries and nanoinclusions) is a viable way to manipulate the thermal conductivity.

## Conclusion

In summary, the phonon transport properties of borophene have been investigated through solving the Boltzmann transport equation based on the first-principle calculations. At room temperature, the lattice thermal conductivity of borophene is predicted to be 14.34 W/mK (error is about 3%) which is significantly reduced as compared to that of suspended graphene (about 3500 W/mK). The contributions from different phonon modes to the thermal transport are extracted, and some high frequency phonon modes unexpectedly contribute to over 70% of the total thermal conductivity of borophene at room temperature. This is quite deviating from the commonly believed that the thermal transport is usually dominated by the low frequency acoustic phonon modes for most of suspended 2D materials. There are two reasons for understanding such predominant contribution of high frequency phonon modes on the overall thermal transport of borophene. (1). The interaction of high frequency phonon modes with three acoustic phonon modes and among themselves results in the relatively high group velocity. (2). The intrinsic frequency gap (energy gap, around the K point) among the high frequency phonon modes will restrict the anharmonic phonon-phonon scattering and thus lead to the large phonon relaxation time. Meanwhile, the anharmonic scattering phase space is further analyzed, which reveals that the scattering between FA and other phonon modes such as 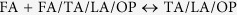 is the major phonon process channel. Finally, the vibrational characteristic of some typical phonon modes and the accumulative thermal conductivity as a function of MFP is presented in this paper as well. These findings provided in this paper not only elucidate on the thermal transport properties of borophene, but also underpin the potential application in the thermal management community.

## Model and Method

Our first-principles calculations are performed based on the density functional theory (DFT) as implemented in the Vienna ab initio simulation package (VASP)^34^. The Perdew-Burke-Ernzerhof of generalized gradient approximation is adopted as the exchange correlation function^35^,^36^,^37^. The kinetic-energy cut off of the plane-wave basis is chosen as 500 eV and a Monkhorst–Pack k-mesh of 15 × 15 × 1 is utilized to sample the reciprocal space of the primitive unit cell of borophene. A large vacuum layer of 15 Å is used to avoid the interactions between periodic borophene layers originated from the periodic boundary condition. The geometric structure of borophene is fully relaxed through the conjugate gradient algorithm until the maximum force on each atom is smaller than 1 × 10^−6^  eV/Å.

For the calculation of phonon dispersion and the harmonic second-order interatomic force constants (2^nd^ IFCs), a supercell (4 × 4 × 1) containing 128 boron atoms is constructed, and the corresponding numbers of k-mesh are accordingly scaled down compared with the case of unit cell calculation. These are performed in the harmonic approximation via means of finite displacement method implemented in the PHONOPY package^38^. Besides the harmonic IFCs, the anharmonic third-order IFCs (3^rd^ IFCs) which including the information of phonon-phonon scatterings are also necessary for the calculation of phononic thermal conductivity. The same 4 × 4 × 1 supercell is employed to obtain the anharmnic IFCs, and the cutoff of interaction range is taken into account up to forth nearest neighbors. Meanwhile, we use the Lagrangian multiplier method to enforce the translational invariance constraint of 3^rd^ IFCs^39^. Based on the obtained 2^nd^ and 3^rd^ IFCs, the phonon transport properties of borophene could be calculated by iteratively solving the phonon Boltzmann transport equation (PBTE) as implemented in the ShengBTE package^40^.

## Additional Information

**How to cite this article:** Xiao, H. *et al*. Lattice thermal conductivity of borophene from first principle calculation. *Sci. Rep.* **7**, 45986; doi: 10.1038/srep45986 (2017).

**Publisher's note:** Springer Nature remains neutral with regard to jurisdictional claims in published maps and institutional affiliations.
